# Ultrasound derived deep learning features for predicting axillary lymph node metastasis in breast cancer using graph convolutional networks in a multicenter study

**DOI:** 10.1038/s41598-025-13086-0

**Published:** 2025-07-30

**Authors:** Enock Adjei Agyekum, Wentao Kong, Doris Nti Agyekum, Eliasu Issaka, Xian Wang, Yong-zhen Ren, Gongxun Tan, Xuan Jiang, Xiangjun Shen, Xiaoqin Qian

**Affiliations:** 1 Department of Ultrasound, Jiangsu University Affiliated People’s Hospital, Zhenjiang, 212002 China; 2 School of Computer Science and Communication Engineering, Jiangsu University, Zhenjiang, Jiangsu Province China; 3 Northern Jiangsu People’s Hospital Affiliated to Yangzhou University, Yangzhou, China; 4 Northern Jiangsu People’s Hospital, Yangzhou, Jiangsu Province China; 5 The Yangzhou Clinical Medical College of Xuzhou Medical University, Yangzhou, Jiangsu Province China; 6 Department of Medical Laboratory Technology, University of Cape Coast, Cape Coast, Ghana; 7 College of Engineering, Birmingham City University, Birmingham, B4 7XG UK

**Keywords:** Axillary lymph node metastasis, Breast cancer, Deep learning features, Recursive feature elimination, Graph convolutional network, Cancer, Medical research, Oncology

## Abstract

The purpose of this study was to create and validate an ultrasound-based graph convolutional network (US-based GCN) model for the prediction of axillary lymph node metastasis (ALNM) in patients with breast cancer. A total of 820 eligible patients with breast cancer who underwent preoperative breast ultrasonography (US) between April 2016 and June 2022 were retrospectively enrolled. The training cohort consisted of 621 patients, whereas validation cohort 1 included 112 patients, and validation cohort 2 included 87 patients. A US-based GCN model was built using US deep learning features. In validation cohort 1, the US-based GCN model performed satisfactorily, with an AUC of 0.88 and an accuracy of 0.76. In validation cohort 2, the US-based GCN model performed satisfactorily, with an AUC of 0.84 and an accuracy of 0.75. This approach has the potential to help guide optimal ALNM management in breast cancer patients, particularly by preventing overtreatment. In conclusion, we developed a US-based GCN model to assess the ALN status of breast cancer patients prior to surgery. The US-based GCN model can provide a possible noninvasive method for detecting ALNM and aid in clinical decision-making. High-level evidence for clinical use in later studies is anticipated to be obtained through prospective studies.

## Introduction

The second most common cause of cancer-related death in women is breast cancer, a serious global health problem^1,2^. It accounts for approximately 23% of all cancers in women^3^. Early identification and efficient treatment can significantly increase the survival rate of female patients with breast cancer^4^. Axillary lymph nodes (ALNs) are the most prevalent site of metastasis. These nodes are essential for pathological staging, prognosis, and therapy planning. This involves determining whether to provide neoadjuvant or adjuvant treatment and assisting patients in planning their surgical procedures^5^. Determining axillary lymph node metastasis (ALNM) accurately in breast cancer patients is crucial for determining their prognosis and course of treatment^6^. ALN dissection and sentinel lymph node (SLN) biopsy are the current approaches for detecting the metastatic status of ALNs^7–9^. However, both procedures are invasive and carry a risk of postoperative morbidity, including edema of the upper limbs and numbness in the arms^10,11^. Thus, the best course of therapy for individuals with breast cancer would involve the use of a reliable, noninvasive method to predict ALNM.

The most common method for evaluating ALNs in patients with breast cancer is ultrasound (US), which has been extensively utilized to define breast lesions preoperatively^12^. However, the performance is unsatisfactory^13,14^. To avoid needless ALN dissection, it is critical to perform a more accurate examination before surgery to detect ALNM in breast cancer patients. Radiomics provides the capacity to automatically extract several quantitative features from medical images that may be difficult for the human eye to recognize^15,16^. The limitations of manually constructed features are centered on the requirement for human labeling and their inability to adapt exactly to a particular task^17^. Deep learning^16^, on the other hand, is a novel technique capable of autonomously revealing several layers of representation adapted to specific prediction tasks via end-to-end learning. Graph neural networks (GNNs) are a type of deep learning algorithm designed to infer graph-described data^18^. GNNs are neural networks that can be directly applied to graphs and provide a simple approach to accomplish prediction tasks at the node, edge, and graph levels^19^. Graph-based models have demonstrated promising outcomes in numerous computer vision applications where nodes and their interactions are integrated^20^.

There have been several studies on the application of the GNN in breast cancer diagnosis^18–21^; however, there is currently no research on the use of the GNN in breast cancer US imaging to predict ALNM in patients with breast cancer. In this study, on the basis of US deep learning features, a graph convolutional network (GCN) model was created to predict ALNM in breast cancer patients.

## Materials and methods

### Patients

**Fig. 1:**
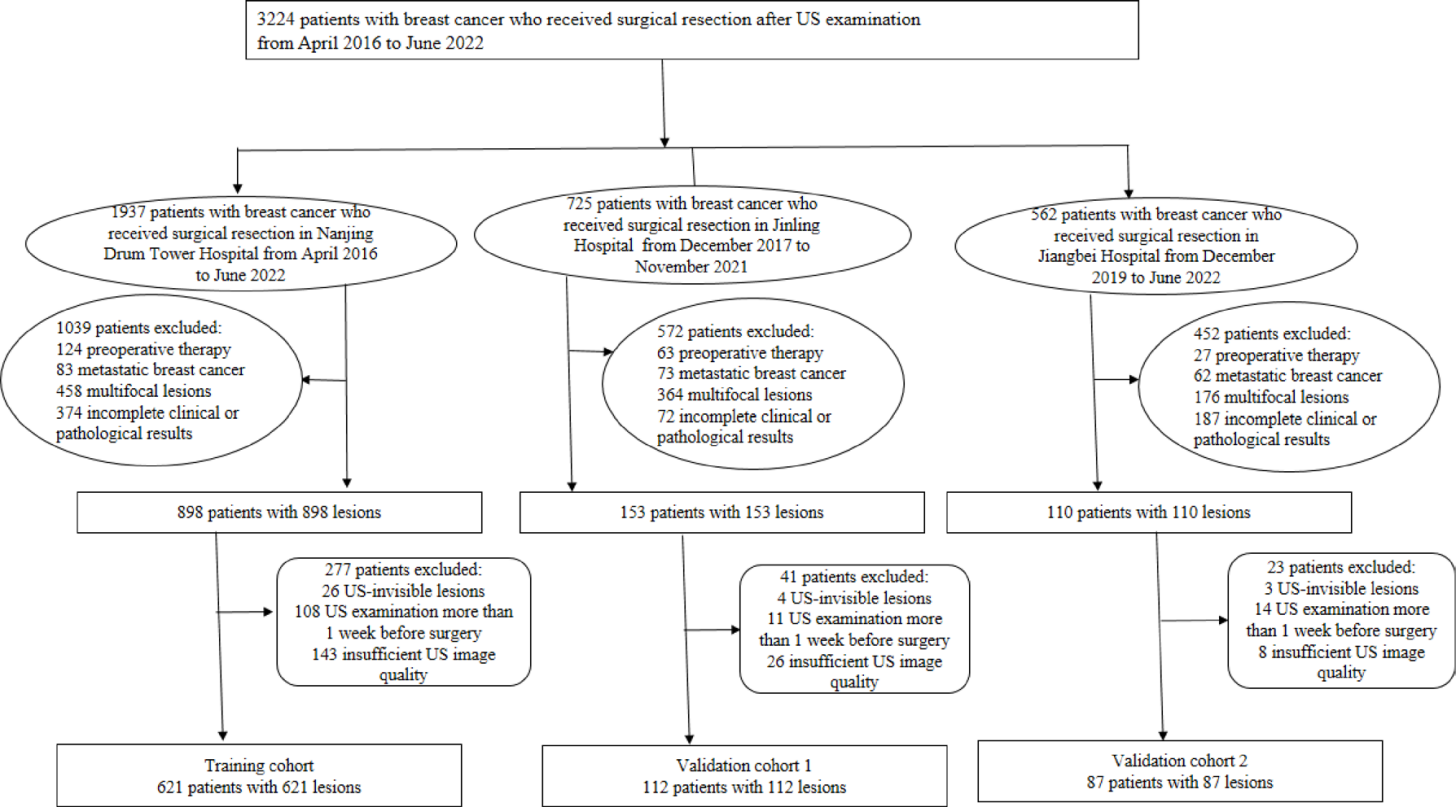
Schematic diagram of patient selection. US, ultrasound.

This research conformed to the ethical criteria of the Declaration of Helsinki and was approved by the institutional review board of Nanjing Drum Tower Hospital. The retrospective nature of this study allowed for the waiver of informed consent requirements. Table 1 displays the inclusion and exclusion criteria. This research included 820 individuals from three hospitals who had histologically confirmed primary breast cancer. The method of patient recruitment is outlined in the flowchart presented in Fig. 1. The training cohort consisted of 621 breast cancer patients who were retrospectively recruited from Nanjing Drum Tower Hospital between April 2016 and June 2022. Validation Cohort 1 consisted of 112 patients who were recruited from Jinling Hospital between December 2017 and November 2021. Validation Cohort 2, consisting of 87 patients from Jiangbei Hospital, was enrolled between December 1, 2019, and June 30, 2022.

**Table 1:** Inclusion and exclusion criteria.

Inclusion and exclusion criteria
Inclusion criteria
1. women with histologically diagnosed unifocal breast cancer
2. patients with confirmed ALN status by ALN dissection/SLN biopsy
3. patients received ultrasound examination within 1 week before surgery
Exclusion criteria
1. patients received neoadjuvant radiotherapy, chemotherapy, or other therapies preoperatively
2. patients with ultrasound-invisible or non-mass-type lesions
3. patients with multifocal lesions or insufficient image quality
4. patients with metastatic breast cancer
5. Patients with incomplete clinical or histopathological information.

ALN, axillary lymph node; SLN, sentinel lymph node.

**Table 2:** Patient characteristics of the Training and Validation Cohorts.

Characteristic training cohort validation cohort 1 validation cohort 2
***Age(years)*** *54.50 ± 11.51 55.19 ± 10.65 53.93 ± 11.94*
***Body mass index***
*<25 237 (38.16%) 36 (32.14%) 26 (29.89%)*
*25–30 172 (27.70%) 36 (32.14%) 27 (31.03%)*
*>30 174 (28.02%) 35 (31.25%) 32 (36.78%)*
*Not applicable 38 (6.12%) 5 (4.46%) 2 (2.30%)*
***US size (cm)*** *2.24 ± 1.06 2.26 ± 1.12 2.17 ± 1.21*
***BI-RADS category***
*4 A 172 (27.70%) 18 (16.07%) 6 (6.90%)*
*4B 144 (23.19%) 35 (31.25%) 12 (13.79%)*
*4 C 133 (21.42%) 8 (7.14%) 12 (13.79%)*
*5 172 (27.70%) 51 (45.54%) 57 (65.52%)*
***Nuclear grade***
*I 96 (15.46%) 5 (4.46%) 13 (14.94%)*
*II 298 (47.99%) 67 (59.82%) 49 (56.32%)*
*III 227 (36.55%) 40 (35.71%) 25 (28.74%)*
***Tumor location***
*UOQ 130 (20.93%) 21 (18.75%) 25 (28.74%)*
*LOQ 61 (9.82%) 12 (10.71%) 6 (6.90%)*
*UIQ 163 (26.25%) 38 (33.93%) 25 (28.74%)*
*LIQ 66 (10.63%) 26 (23.21%) 13 (14.94%)*
*Central 201 (32.37%) 15 (13.39%) 18 (20.69%)*
***Tumor classification***
*Noninvasive 59 (9.51%) 4 (3.57%) 7 (8.05%)*
*Carcinoma*
*Invasive carcinoma*
*No special type 489 (78.74%) 97 (86.61%) 71 (81.61%)*
*Special type 43 (6.92%) 8 (7.14%) 6 (6.90%)*
*Rare carcinoma 30 (4.83%) 3 (2.68%) 3 (3.45%)*
***HER2***
*Positive 520 (83.73%) 34 (30.36%) 66 (75.86%)*
*Negative 101 (16.26%) 78 (69.64%) 21 (24.14%)*
***ER***
*Positive 451 (72.62%) 83 (74.11%) 64 (73.56%)*
*Negative 170 (27.38%) 29 (25.89%) 23 (26.44%)*
***Ki-67***
*Positive 514 (82.77%) 90 (80.36%) 66 (75.86%)*
*Negative 107 (17.23%) 22 (19.64%) 21 (24.14%)*
***PR***
*Positive 402 (64.73%) 78 (69.64%) 52 (59.77%)*
*Negative 219 (35.27%) 34 (30.36%) 35 (40.23%)*
***Surrogate subtype***
*Luminal A-like 248 (39.94%) 16 (14.29%) 6 (6.90%)*
*Luminal B-like 216 (34.78%) 71 (63.39%) 58 (66.67%)*
*HER2 127 (20.45%) 11 (9.82%) 20 (22.99%)*
*Overexpression*
*Triple-negative 30 (4.83%) 14 (12.50%) 3 (3.45%)*
***ALN metastasis***
*Positive 215 (34.62%) 45 (40.18%) 35 (40.23%)*
*Negative 406 (65.38%) 67 (59.82%) 52 (59.77%)*
***US-ALN***
*Suspicious 242 (38.97%) 37 (33.04%) 44 (50.57%)*
*Non-suspicious 379 (61.03%) 75 (66.96%) 43 (49.43%)*

US ultrasound, BMI body mass index, UOQ upper outer quadrant, LOQ lower outer quadrant, UIQ upper inner quadrant, LIQ lower inner quadrant, ER estrogen receptor, PR progesterone receptor, HER2 human epidermal growth factor receptor-2, US-ALN axillary lymph nodes status reported by axillary ultrasound.

Every patient underwent a standard two-dimensional US examination. The patients were divided into two groups, those with ALNM and those without ALNM, on the basis of pathological results. Preoperative clinical factors for the study included clinicopathological features as well as US results of the breast and axilla. Age, body mass index (BMI), progesterone receptor (PR) status, estrogen receptor (ER) status, Ki-67 expression, human epidermal growth factor receptor 2 (HER2) expression, tumor categorization, nuclear grade, and surrogate subtype were among the clinicopathological variables examined. The tumor location, breast lesion US size, Breast Imaging Reporting and Data System (BI-RADS) category, and ALN status, as revealed by axillary ultrasound (US-ALN), were among the US findings of the breast and axilla.

### Ultrasound examination

Before surgery, all patients underwent regular axillary and breast US, which was carried out by highly skilled radiologists from three hospitals with at least five years of breast US expertise using a Siemens S3000 with a linear transducer operating at 4–9 MHz, Philips IU22 with a 6–12 MHz linear transducer, Philips EPIQ5 with a 6–12 MHz linear transducer, GE Healthcare LOGIQ E9 with a 6–15 MHz linear transducer, Siemens S2000 with a 4–9 MHz linear transducer, and MyLab twice with a 5–13 MHz linear transducer.

The patients were kept in a supine position with their ipsilateral arms raised to a minimum of 90 degrees and their corresponding hands behind their heads while breast and axillary US were performed. This made it possible to examine the breast and axilla seamlessly and comfortably. The application pressure was chosen to ensure that patients would not experience discomfort and that the anatomy could be seen on the B-mode image without artifacts. The breast was scanned in the transverse, sagittal, and radial scan directions. Essentially, the examiner chose the scan orientations so that the whole breast could be seen and scanned via an overlapping approach. All of the images were stored in the Picture Archiving and Communication Systems for further research. For each patient, one single image of the target breast mass was selected at the maximum diameter plane for further analysis.

### Data preprocessing and feature extraction

**Fig. 2:**
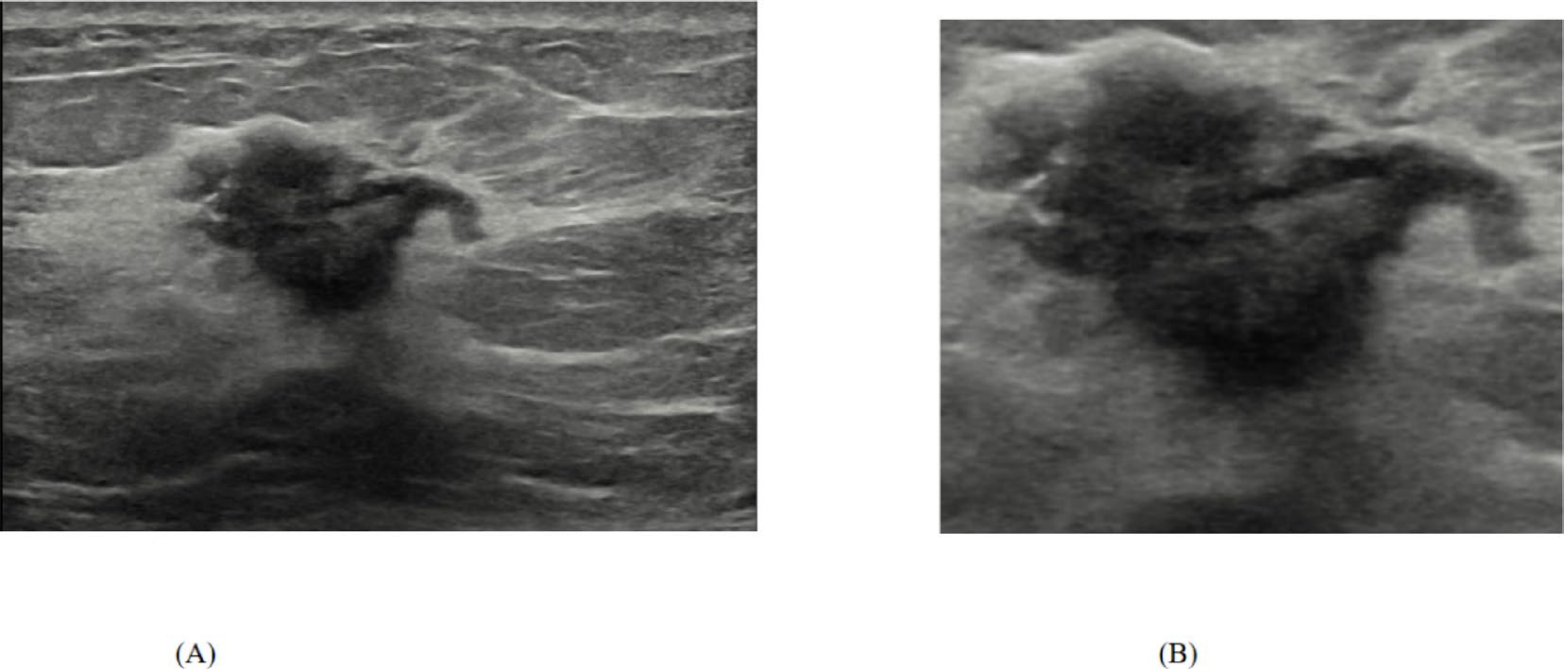
A shows the original breast ultrasound image. B displays the corresponding bounding box annotation used to localize the lesion region for subsequent analysis.

The imaging repository was reviewed to locate all the breast cancer US images, and the ALNM status was correlated with the US imaging data. Prior to preprocessing, grayscale conversion was applied to all B-mode US images to eliminate unnecessary image channels. The masses were detected by bounding box labels, and a radiologist with eight years of experience in the US confirmed the annotations. The rectangular areas of interest (ROIs) were derived from the original US images (Fig. 2). Cropped US images were resized, padded, and normalized to 224 × 224 pixels, retaining their original aspect ratios, to reduce the impact of irrelevant background information. A pixel boundary was created around the lesion zone to capture part of the nearby region, which might provide useful information and prevent inadequate mass extraction. All preprocessing steps were conducted in Python (version 3.10.12) via Keras preprocessing.

In this study, we used ResNet50 as the basic model for obtaining deep learning features. Previous studies have demonstrated the effectiveness of ResNet50 in yielding high-quality deep learning features^22,23^. Building upon AlexNet’s groundwork, ResNet was created and enhanced, showing benefits such as identity mapping and an impressive depth of 152 layers. Nevertheless, the challenge of vanishing or exploding gradients arises as the network depth increases^24–26^.

To address this issue, methods such as normalized initialization and the incorporation of intermediate normalization layers have been introduced^25–28^. Despite these efforts, as network layers stack, the accuracy of the training dataset may plateau or even decline, a phenomenon known as degradation. Notably, this decline is not caused by overfitting; rather, it is exacerbated by the addition of layers to the model^29,30^. ResNet tackles this issue by employing a deep residual learning framework, effectively mitigating degradation^31^. In this study, transfer learning was used to fine-tune all the weights and biases, considerably reducing the training time. The parameters were pretrained on the ImageNet dataset before being loaded and retrained on our dataset. Finally, the original classifier for the ImageNet classes was replaced with a binary classifier, yielding a class probability vector ranging from 0 to 1 as the prediction result for each patient.

The network was trained from scratch using the cross-entropy loss function and the Adam optimizer, with a learning rate of 0.0001 and a batch size of 32. The implementation of the training and validation codes utilized PyTorch version 2.2.2 + cu118 and Keras version 2.10.0. The model underwent 200 epochs of training to prevent overfitting.

Data augmentation was applied for the training cohort to reduce the potential bias caused by the limited number of images in the training procedure^32^. US images in the training cohort were augmented through several random transformations, including image flipping horizontally and vertically, cropping, and rotation, which could increase the training data pool and decrease the overfitting of the generated deep learning model. In the ResNet50 model, the fully connected layer and softmax layer were removed, and the output values of the nodes in the last layer were used as the deep learning features. Ultimately, the ResNet50 model extracted 2048 deep learning features from each breast cancer US image.

### Feature selection

Because it is straightforward to implement and use, recursive feature elimination (RFE) is widely used in machine learning to choose the most important features from a training dataset for predicting the target variable^33^. RFE boosts model effectiveness by prioritizing these relevant characteristics, reducing overfitting, and improving interpretability^33^. The procedure involves gradually removing the least important elements until the target number of features is achieved, ultimately resulting in building a model with the remaining features^34^.

To prevent bias and overfitting, the retrieved deep learning features were normalized with a standard scalar. Because the feature space is highly dimensional, the number of features must be minimized to avoid the interference of a large number of redundant features in the data analysis, which has an influence on model creation and increases computational costs.

To reduce feature redundancy and ensure statistical independence among variables, we applied Spearman’s rank correlation to the training feature matrix. A pairwise correlation matrix was computed, and features were considered redundant if their absolute Spearman correlation coefficient exceeded 0.85. In such cases, one feature from each highly correlated pair was removed. This approach effectively minimized row-wise spatial dependencies in the feature matrix, resulting in a set of features that were substantially independent and more informative for model training. To identify the most informative subset of ALNM-related predictive features for classification, we employed Recursive Feature Elimination with Cross-Validation (RFECV). A linear Support Vector Machine with a regularization parameter *C = 1.* The RFECV algorithm recursively eliminated the least important features based on the SVM’s weights, while evaluating model performance at each step using 10-fold stratified cross-validation to preserve class distribution.

**Fig. 3:**
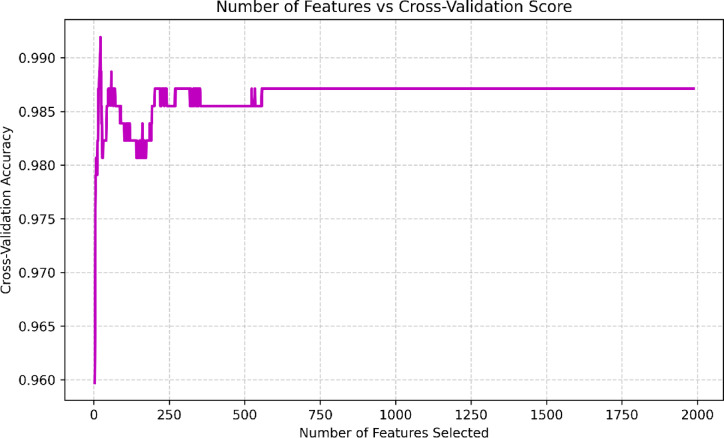
Recursive feature elimination (RFE) with 10-fold stratified cross-validation; number of features selected vs. cross-validation score.

The minimum number of features to retain was set to 3, ensuring that even under maximum reduction, the model maintained a minimal yet potentially effective feature set. The selection process was driven by classification accuracy as the scoring metric, and the final feature set was determined at the point where cross-validation performance was optimized (Fig. 3). This approach enabled robust feature ranking while mitigating the risk of overfitting.

### Construction of the graph

A feature table of 621 rows and 24 columns for the training cohort, 112 rows and 24 columns for validation cohort 1, and 87 rows and 24 columns for validation cohort 2 was initially constructed to construct the graph. The 24 columns included 22 RFE-selected features, a unique ID for each row, and the target class. Each row was treated as one separate node (*v*). The cosine similarity between rows was computed to create an edge (*E*) table.

On the basis of each image’s unique attributes, this quantifies the connections between the images. For example, nodes 1 to 2, nodes 1 to 3, nodes 1 to 621, and nodes 2 to 3, nodes 2 to 4, and nodes 2 to 621 are examples of how the link is determined by computing the similarity scores of each row. Each time, the cosine similarity between two nodes is calculated, and a relationship between a specific node and all other nodes is discovered.

With the set of nodes (*v*_*n*_) and edges (*e*_*m*_), a graph *G* = (*V*,* E*) is generated where *v*_*n*_ ∈ *V* and *e*_*n*_ ∈ *E*, *n*, *m* denote the number of nodes and edges, respectively. In a graph, the edges are constructed as *eij* = (*vi*,* vj*), which represents the relationship between nodes *vi* and *vj*. An adjacency matrix (A) is also generated from the graph (G) with n × n dimensions. If *Aij* = 1, there is an edge between two nodes (*eij* ∈ *E*), and *Aij* = 0 if *eij* ∉ E.

**Fig. 4:**
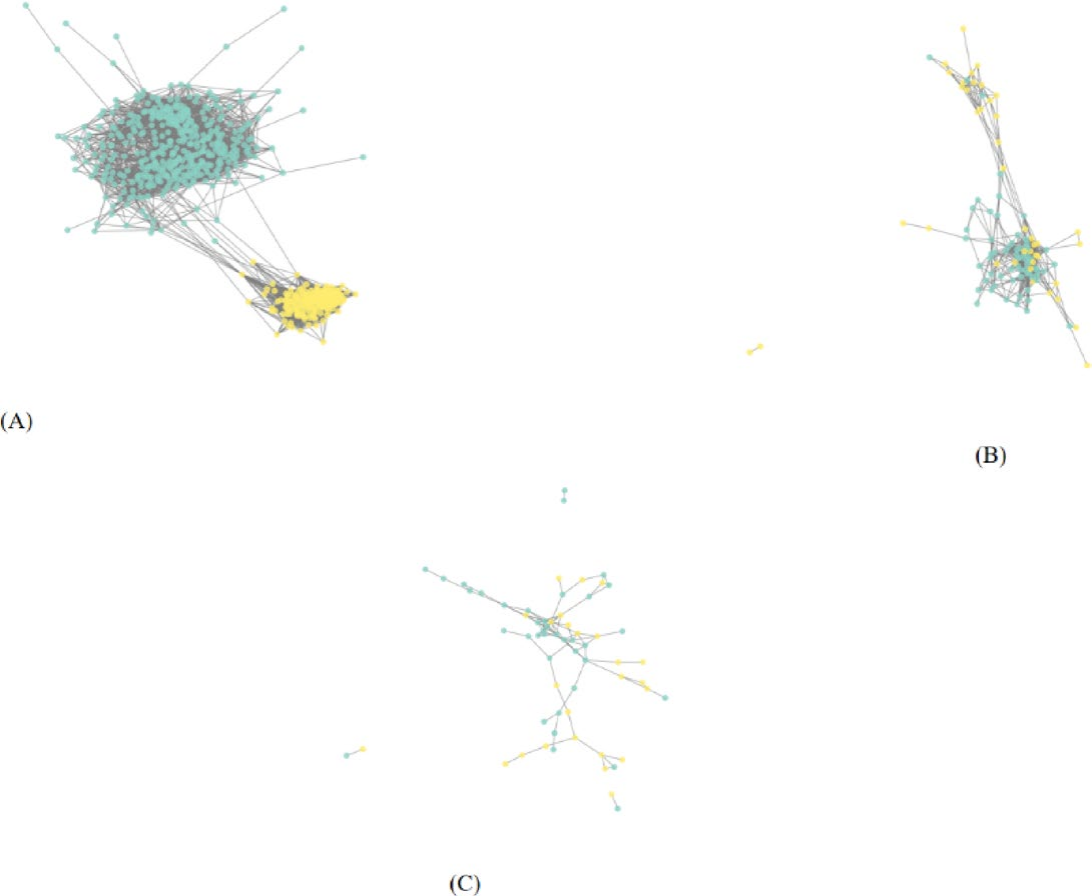
A visualization of the graph. (**A**) Training cohort (**B**) Validation cohort 1 (**C**) Validation cohort 2.

The 22 chosen features, excluding the unique target classes, make up the graph’s feature vectors X, where *Xv* stands for the feature vector of the particular node (*v*)^35^. The relationships between images serve as the foundation for the created graph. The resulting graph is large and complex; therefore, only a small portion is illustrated in Fig. 4 using a threshold 0.60. Graph visualization was performed using NetworkX and Matplotlib by plotting only the connected components, enabling inspection of the graph structure and the distribution of classes.

### Construction of the model

In this study, we used the GCN to create our model, which captures the link between nodes by performing convolution operations on nodes and their neighbors to enable feature extraction and representation learning from graph structure data. The GCN integrates convolution operations into a graph structure and is one of the most important GNNs at the moment^36,37^. The GCN is developed from graph signal processing, and a filter is introduced to construct graph convolution, which can be viewed as reducing noise through a filter to obtain the classification result of the input signal^38^.

The GCN model consisted of two graph convolutional layers with ReLU activation functions, followed by a dropout layer and a fully connected output layer. The graph convolution operation in each layer is defined as follows:

1 $$\:H^{(1+1)}\:=\sigma \:\:(\tilde D^{-1/2} \: \tilde{A} \tilde D^{-1/2} \:H^{(l)}W^{(l)})$$

**Fig. 5:**
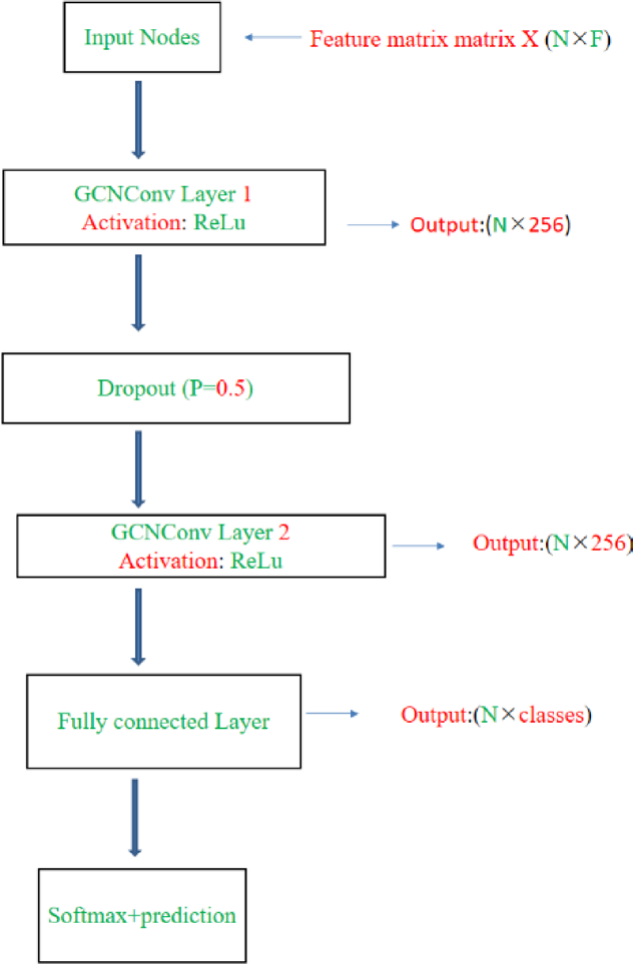
Architecture of the proposed GCN model.

Where: $H^{(1)} \in {R}^{NxFl}$ is the matrix of node features at layer *l* (with *H*^(0)^ = *X*, the input features), *Ã* = *A + I* is the adjacency matrix with added self-loops, $\tilde{D}$ is the diagonal degree matrix of *Ã*, *W*^(1)^ is the trainable weight matrix at layer *l*, *σ (.)* is the activation function (ReLU in this case).The final output from the second GCN layer is passed through a fully connected layer: Where Z contains the class probabilities for each node and W^(fc)^ is the weight matrix of the output layer. The GCN architecture is depicted in Fig. 5.

2 $$\:Z = {\rm Softmax}\:(H^{(2)}\:W^{(fc)})$$

The ALNM status of breast cancer patients was represented with one-hot encoding as the label. During the training phase, the constructed graphs were fed into the network to update the model parameters. The network outputs serve as classification results, and the loss function is determined by computing the cross-entropy between the outputs and labels.

**Fig. 6:**
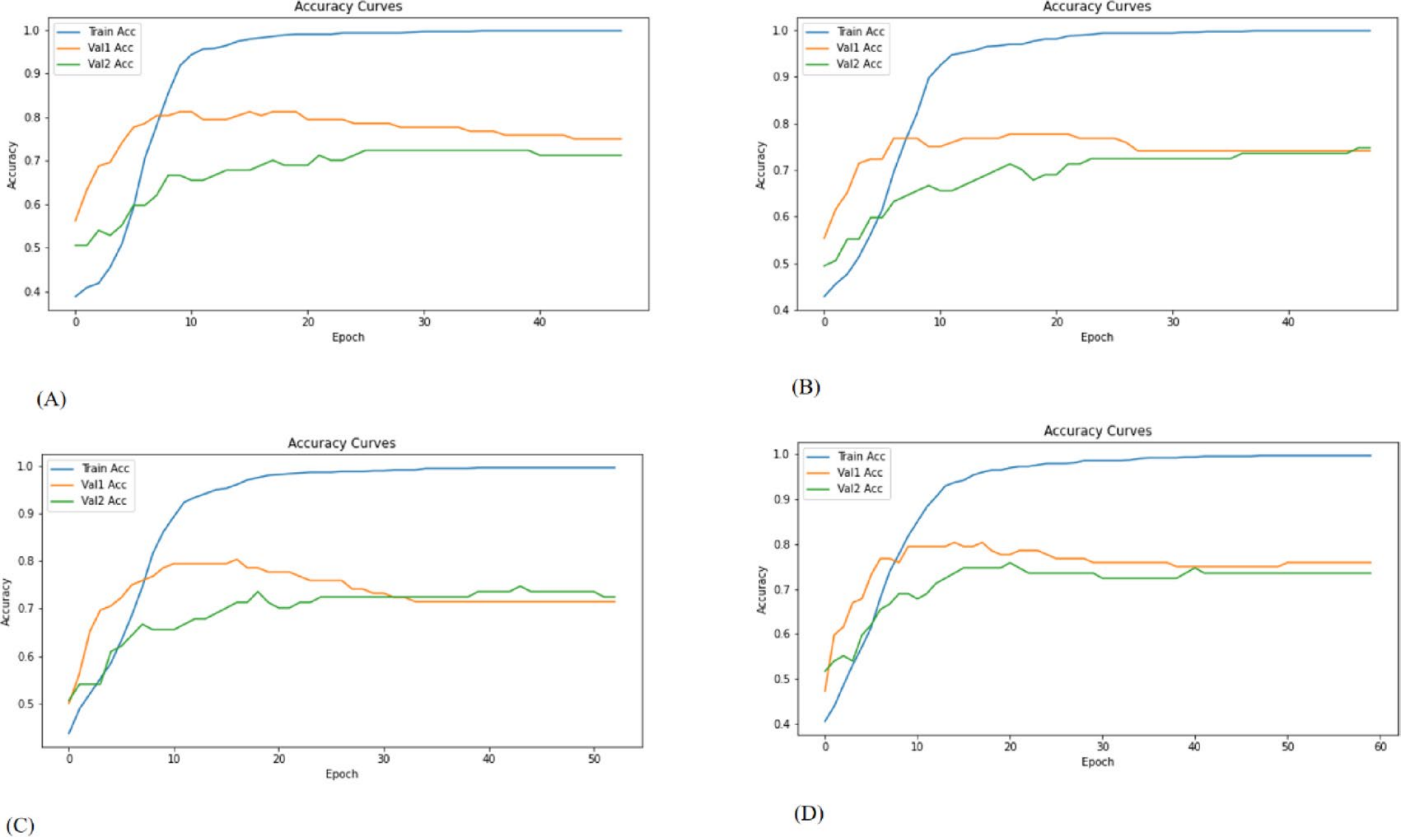
Accuracy curves of the models. Blue represents training cohort and yellow and green curves represent the validation cohort 1 and validation cohort 2, respectively. (A)Threshold_0.60 (B) Threshold_0.70 (C) Threshold_0.80 (D) Threshold_0.95.

**Fig. 7:**
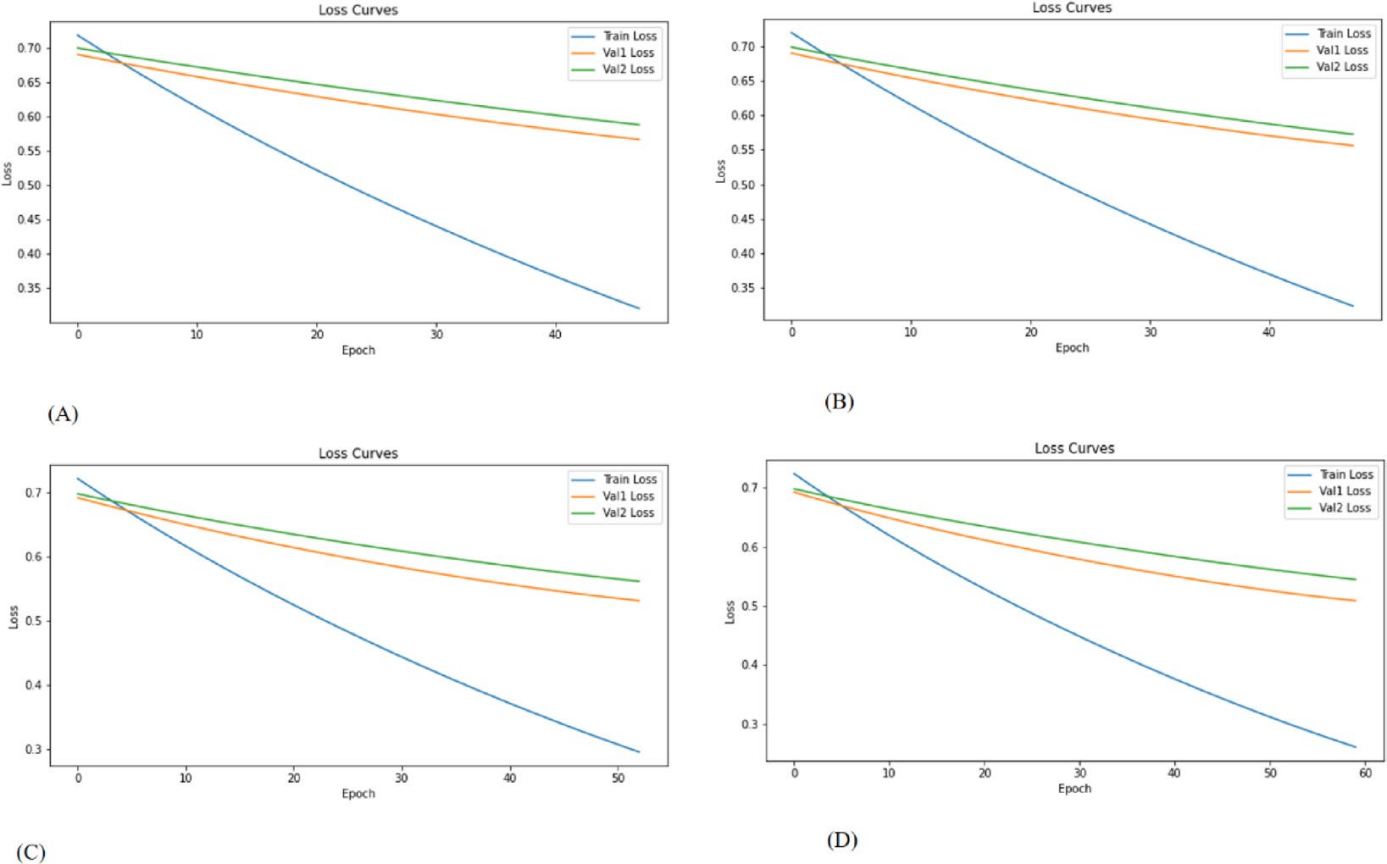
Loss curves of the models. Blue represents training cohort and yellow and green curves represent the validation cohort 1 and validation cohort 2, respectively. (A)Threshold_0.60 (B) Threshold_0.70 (C) Threshold_0.80 (D) Threshold_0.95.

Training was performed using the Adam optimizer with a learning rate of 0.0001 and a batch size of 32. We chose a batch size of 32 and a learning rate of 0.0001 based on previous research and their balance of computing efficiency and training stability. A learning rate of 0.0001 ensures smooth convergence, while the batch size of 32 allows for steady gradient updates without consuming too much memory. An early stopping strategy with patience was employed based on the validation loss to mitigate overfitting. The model was trained for up to 500 epochs on a graph constructed from the training set, with evaluation performed on two separate validation graphs (Validation Cohort 1 and Validation Cohort 2).All graph operations and GCN layers were implemented using PyTorch Geometric (PyG), built on PyTorch version 2.2.2, and integrated with Keras (version 2.10.0) for compatibility in preprocessing pipelines. Python version 3.10.12 was used throughout. Additionally, training and validation loss and accuracy curves were plotted to assess model convergence and generalization performance (Figs. 6 and 7).

**Fig. 8:**
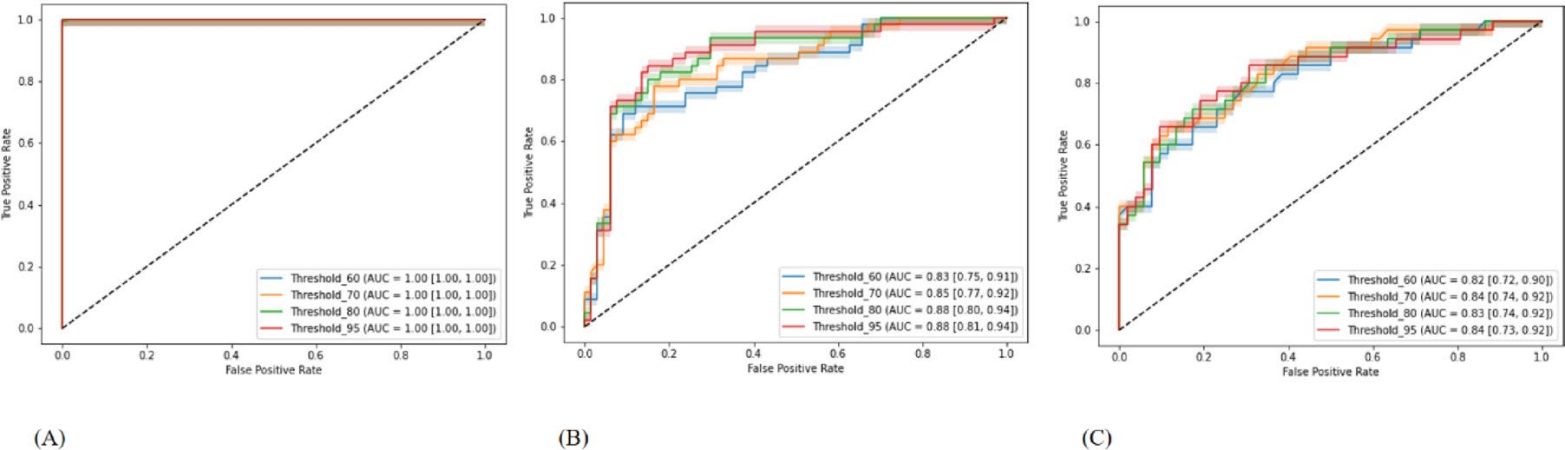
ROC curves of the models employing different thresholds. ROC, receiver operating characteristic. (A) Training cohort (B) Validation cohort 1 (C) Validation cohort 2 *Note: Threshold labels were expressed in percentage format (e.g.*,* “Threshold_95” corresponds to a threshold value of 0.95)*.

Several thresholding experiments were conducted to improve the model’s performance via cosine similarity thresholds of 0.60, 0.70, 0.80, and 0.95. The goal was to determine which threshold yielded the best results and whether a shallower graph or fewer edges resulted in higher accuracy.

### Evaluation metrics employed in this study

The models’ performance on both the training and validation datasets was evaluated using metrics such as the area under the curve (AUC), confusion matrix, precision, sensitivity, specificity, negative predictive value (NPV), positive predictive value (PPV), accuracy, and other standard clinical statistics.

The ROC curve plots the true positive rate (sensitivity) against the false positive rate (1 – sensitivity), and the AUC can be calculated. The accuracy, in the context of ALNM prediction, gauges the model’s overall accuracy in predicting the presence or absence of ALNM in individuals with breast cancer. The accuracy can be calculated from Eq. (3)^39^. Recall or sensitivity (Eq. 4) for ALNM prediction represents the ratio of correctly predicted cases of ALNM out of all actual cases of ALNM based on the true positive rate, showing a model’s capacity to correctly identify all people who have ALNM^40^.

In the context of ALNM prediction, specificity (Eq. 5) is described in a variety of ways, including a model’s capacity to detect true negatives, being based on the true negative rate, and properly identifying those who do not have ALNM. These evaluation metrics play a crucial role in assessing the efficiency and effectiveness of machine learning models. In Eqs. 3, 4, 5, 6, and 7, *TP* represents the number of true positive predictions (correctly predicted positive ALNM), *FP* represents the number of false positive predictions (incorrectly predicted positive ALNM), *TN* represents the number of true negative predictions (correctly predicted negative ALNM), and *FN* represents the number of false negative predictions (incorrectly predicted negative ALNM). These metrics provide valuable insights into the accuracy and performance of ALNM predictions.

3 $$accuracy\: = \frac{TP +TN}{TP +FP+TN+ FN}$$

4 $$sensitivity = \frac{TP}{TP+FN}$$

5 $$specificity = \frac{TN}{TN+FP}$$

6 $$PPV = \frac{TP}{TP+FP}$$

7 $$NPV = \frac{FN}{FN+TN}$$

To assess the consistency of four prediction models (threshold_0.60, threshold_0.70, threshold_0.80, and threshold_0.95), model outputs were compared pairwise across the training cohort and two independent validation cohorts. Pearson correlation coefficients (r) were calculated for each model pair to quantify linear correlations while also evaluating model stability and generalizability across datasets. The predicted performance indicators of the models were reported, and the variations in performance between independently trained models were examined using one-way ANOVA.

We evaluated the normality and variance homogeneity assumptions before doing the ANOVA. To determine if variations in AUCs between models were statistically significant, we used the DeLong test, a non-parametric approach for evaluating correlated ROC curves. Pairwise comparisons were made between all four models in each cohort (training, validation 1, validation 2). Heatmaps were used to display P-values from the DeLong tests, indicating statistically significant differences in model discrimination (values < 0.05). Decision Curve Analysis (DCA) was performed to evaluate the net clinical benefit of each model across a range of threshold probabilities. Net benefit incorporates the true positive rate while accounting for false positives and their clinical consequences. DCA plots were generated for each cohort, with the “treat all” and “treat none” strategies included as references.

#### Statistical analysis

Statistical analysis was conducted via Python (version 3.10.12) and IBM SPSS Statistics for Windows version 26.0 (Armonk, New York, USA). To compare differences in categorical characteristics, either Pearson’s chi-square test or Fisher’s exact test was used. For continuous variables with a normal distribution, the independent sample t test was utilized, whereas for variables without a normal distribution, the Mann‒Whitney U test was applied. A two-sided P value < 0.05 was considered indicative of a statistically significant difference.

## Results

### Clinical characteristics

 The clinical characteristics of the 820 breast cancer patients across the three institutions are presented in Table 2. The ALN metastasis rates in the training cohort, validation cohort 1, and validation cohort 2 were 34.6%, 40.2%, and 40.2%, respectively, on the basis of the findings of SLN biopsy or ALN dissection. For each of the three cohorts, the median BMIs were 26.3, 25.7, and 25.2, respectively.

### Diagnostic performance of the model

For every patient, a total of 2048 deep learning features were retrieved. After utilizing Spearman’s rank correlation coefficient, the features were reduced to 1987 features, which were then further analyzed via RFE.

Figure 3 depicts the results of applying RFE to deep learning features. Twenty-two deep learning features were selected for creating the graphs that serve as inputs to the model.

In the training cohort, the model attained accuracies of 1.00 (95% CI: 1.00–1.00), 1.00 (95% CI: 1.00–1.00), 1.00 (95% CI: 0.99–1.00), 1.00 (95% CI: 0.99–1.00) for threshold_0.60, threshold_0.70, threshold_0.80, and threshold_0.95, respectively, and AUCs of 1.00 (95% CI: 1.00–1.00), 1.00 (95% CI: 1.00–1.00), 1.00 (95% CI: 1.00–1.00), and 1.00 (95% CI: 1.00–1.00) for threshold_0.60, threshold_0.70, threshold_0.80, and threshold_0.95, respectively.

In validation cohort 1, the model obtained accuracies of 0.75 (95% CI: 0.67–0.83), 0.74 (95% CI: 0.66–0.82), 0.71 (95% CI: 0.63–0.80), 0.76 (95% CI: 0.68–0.84) for threshold_0.60, threshold_0.70, threshold_0.80, and threshold_0.95, respectively, and AUCs of 0.83 (95% CI: 0.75–0.91), 0.85 (95% CI: 0.77–0.92), 0.88 (95% CI: 0.80–0.94), and 0.88 (95% CI: 0.81–0.94) for threshold_0.60, threshold_0.70, threshold_0.80, and threshold_0.95, respectively.

In validation cohort 2, the model attained accuracies of 0.71 (95% CI: 0.62–0.81), 0.75 (95% CI: 0.66–0.84), 0.72 (95% CI: 0.63–0.82), 0.74 (95% CI: 0.64–0.83) for threshold_0.60, threshold_0.70, threshold_0.80, and threshold_0.95, respectively, and AUCs of 0.82 (95% CI: 0.72–0.90), 0.84 (95% CI: 0.74–0.92), 0.83 (95% CI: 0.74–0.92), and 0.84 (95% CI: 0.74–0.92) for threshold_0.60, threshold_0.70, threshold_0.80, and threshold_0.95, respectively.

**Fig. 9:**
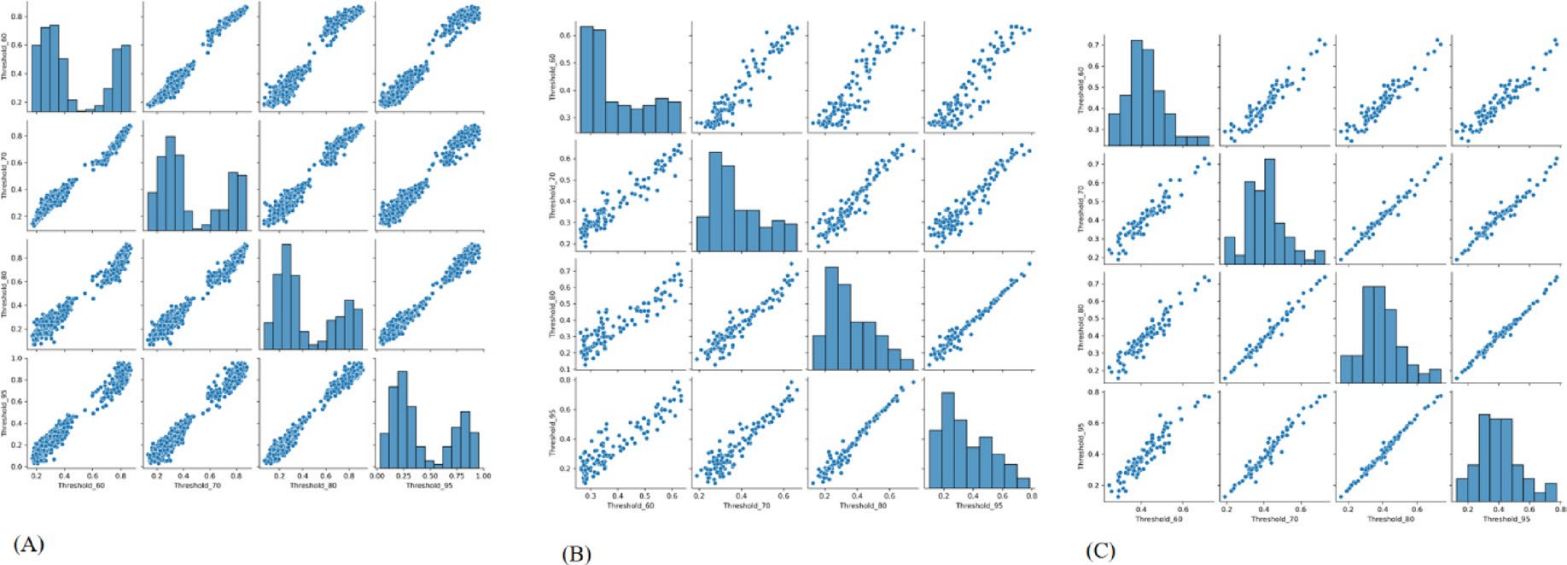
Pairwise comparison of the models’ predictions. Scatter plots illustrate correlations between model outputs within each dataset: (**A**) Training cohort (**B**) Validation cohort 1 (**C**) Validation cohort 2. *Note: Threshold labels were expressed in percentage format (e.g.*,* “Threshold_95” corresponds to a threshold value of 0.95)*.

**Fig. 10:**
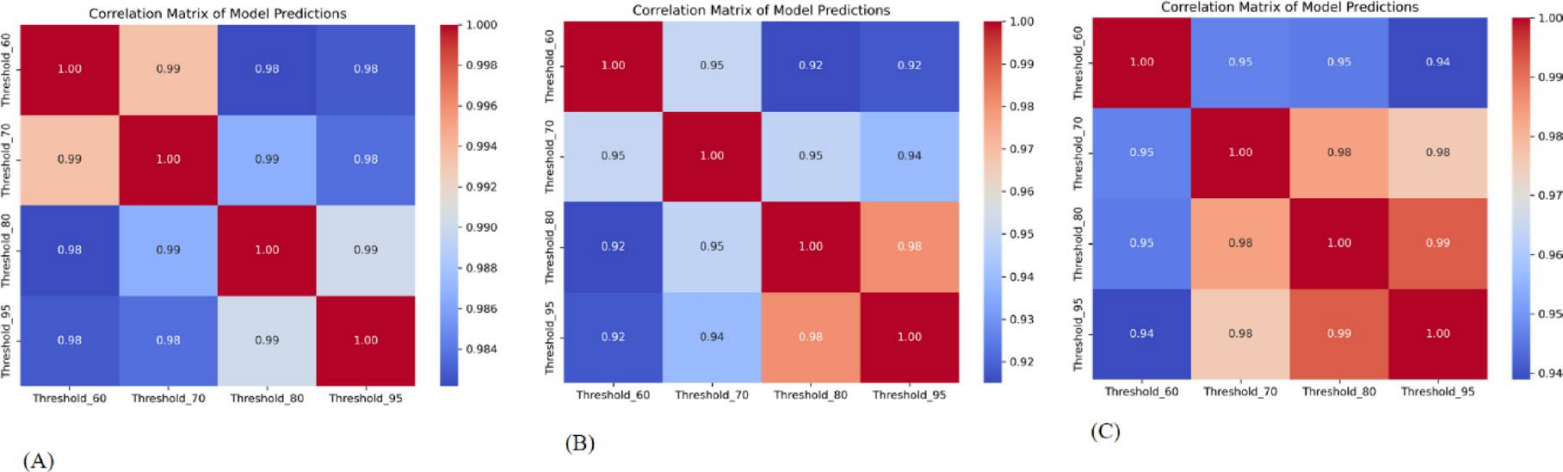
Correlation matrix of the models’ predictions across datasets. (**A**) Training cohort (**B**) Validation cohort 1 (**C**) Validation cohort 2. *Note: Threshold labels were expressed in percentage format (e.g.*,* “Threshold_95” corresponds to a threshold value of 0.95)*.

 The ROC curves for each model are shown in Fig. 8. Table 3 contains extensive data on the models’ prediction performance. Table 4 shows ANOVA, which was used to analyze and compare the predictive performance of our independently trained models. Figures 9 and 10 show pairwise comparisons and analysis of correlation of four predictive models threshold_0.60, threshold_0.70, threshold_0.80, and threshold_0.95, in the training cohort (A), validation cohort 1 (B), and validation cohort 2 (C). Strong correlations were detected between model predictions in the training cohort, with Pearson r values of 0.99 (threshold_0.60 vs. threshold_0.70), 0.98 (threshold_0.60 vs. threshold_0.80), and 0.95. The correlations between the other model pairs varied from 0.95 to 0.98, showing strong consistency.

**Table 3:** Predictive performance of the models for the training and validation cohorts.

ACC (CI) AUC (CI) SEN (CI) SPEC (CI) PPV (CI) NPV (CI)
**Training cohort**
Threshold_60 1.00 (1.00–1.00) 1.00 (1.00–1.00) 1.00 (1.00–1.00) 1.00 (0.99-1.00) 1.00 (0.99-1.00) 1.00 (1.00–1.00)
Threshold_70 1.00 (1.00–1.00) 1.00 (1.00–1.00) 1.00 (1.00–1.00) 1.00 (0.99-1.00) 1.00 (0.99-1.00) 1.00 (1.00–1.00)
Threshold_80 1.00 (0.99-1.00) 1.00 (1.00–1.00) 1.00 (1.00–1.00) 1.00 (0.99-1.00) 1.00 (0.99-1.00) 1.00 (0.99-1.00)
Threshold_95 1.00 (0.99-1.00) 1.00 (1.00–1.00) 1.00 (0.99-1.00) 1.00 (0.99-1.00) 1.00 (0.99-1.00) 1.00 (0.99-1.00)
**Validation cohort 1**
Threshold_60 0.75 (0.67–0.83) 0.83 (0.75–0.91) 0.47 (0.37–0.56) 0.94 (0.90–0.98) 0.84 (0.77–0.91) 0.72 (0.64–0.81)
Threshold_70 0.74 (0.66–0.82) 0.85 (0.77–0.92) 0.44 (0.35–0.54) 0.94 (0.90–0.98) 0.83 (0.76–0.90) 0.72 (0.63–0.80)
Threshold_80 0.71 (0.63–0.80) 0.88 (0.80–0.94) 0.38 (0.29–0.47) 0.94 (0.90–0.98) 0.81 (0.74–0.88) 0.69 (0.61–0.78)
Threshold_95 0.76 (0.68–0.84) 0.88 (0.81–0.94) 0.49 (0.40–0.58) 0.94 (0.90–0.98) 0.85 (0.78–0.91) 0.73 (0.65–0.81)
**Validation cohort 2**
Threshold_60 0.71 (0.62, 0.81) 0.82 (0.72–0.90) 0.40 (0.30–0.50) 0.92 (0.87–0.98) 0.78 (0.69–0.87) 0.70 (0.60–0.79)
Threshold_70 0.75 (0.66–0.84) 0.84 (0.74–0.92) 0.40 (0.30–0.50) 0.98 (0.95-1.00) 0.93 (0.88–0.99) 0.71 (0.61–0.80)
Threshold_80 0.72 (0.63–0.82) 0.83 (0.74–0.92) 0.34 (0.24–0.44) 0.98(0.95- 1.00) 0.92 (0.87–0.98) 0.69 (0.59–0.79)
Threshold_95 0.74 (0.64–0.83) 0.84 (0.73–0.92) 0.43(0.32–0.53) 0.94 (0.89–0.99) 0.83 (0.76–0.91) 0.71 (0.61–0.81)

AUC, area under the curve; ACC, accuracy; CI, confidence interval; SEN, sensitivity; SPEC, specificity; NPV, negative pre-dictive value; PPV, positive predictive value.

**Table 4:** Repeated measures ANOVA results comparing prediction outputs across models.

F Value Num DF Den DF Pr > F
**Training cohort**
165.3911 3.0000 1860.0000 0.0000
**Validation cohort 1**
24.2766 3.0000 333.0000 0.0000
**Validation cohort 2**
18.9294 3.0000 258.0000 0.0000

*The analysis reveals statistically significant differences among the four models in all three cohorts: training cohort (F = 24.28*,*p < 0.001)*,* validation cohort 1 (F = 20.47*,*p < 0.001)*,* and validation cohort 2 (F = 18.93*,*p < 0.001)*,* indicating consistent variation in model prediction performance across datasets.*

In validation cohort 1, inter-model correlations remained high, albeit significantly reduced, with r values of 0.98 (threshold_0.60 vs. threshold_0.70), 0.97 (threshold_0.60 vs. threshold_0.80), and 0.93 (threshold_0.60 vs. threshold_0.95).

In validation cohort 2, greater variation was observed, particularly between threshold_0.60 and threshold_0.95 (*r* = 0.87), though correlations between closer thresholds remained relatively strong (e.g., 0.97 between threshold_0.60 and threshold_0.70, 0.94 between threshold_0.80 and threshold_0.95).These findings demonstrate overall consistency among the models, while highlighting modest divergence in external validation.

**Fig. 11:**
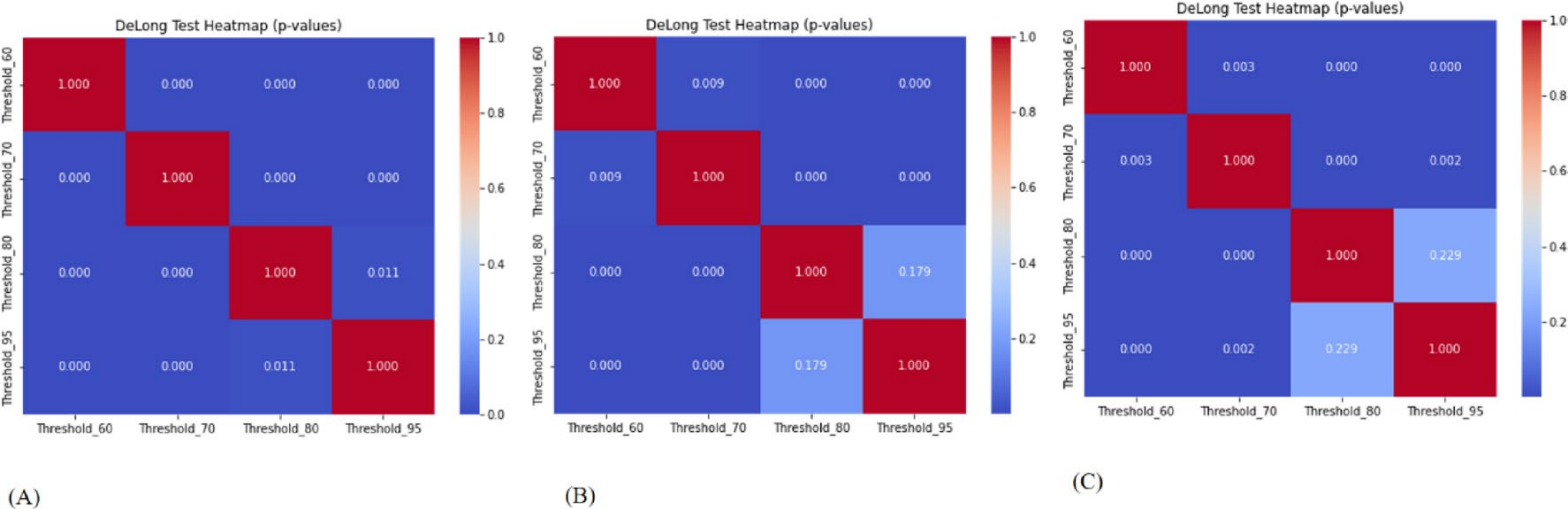
DeLong test heatmaps illustrating pairwise statistical comparisons of model AUCs across models. (**A**) Training cohort (**B**) Validation cohort 1 (**C**) Validation cohort 2. Each cell displays the p-value from DeLong’s test between two models, indicating whether the difference in ROC-AUC is statistically significant. A lower p-value (*p* < 0.05) suggests a meaningful difference in model performance. *Note: Threshold labels were expressed in percentage format (e.g.*,* “Threshold_95” corresponds to a threshold value of 0.95)*.

To assess the four models’ discriminative abilities, pairwise DeLong tests were used to compare AUCs across training, validation cohort 1, and validation cohort 2. Figure 11 (A-C) depicts the results, with p-values shown as a heatmap matrix. In the training cohort (Fig. 11A), all pairwise DeLong comparisons had non-significant p-values (*p* > 0.05), indicating that there were no statistically significant variations in AUCs between the four models. This suggests that the models had equal discriminatory performance on the training dataset, most likely due to identical exposure to training data during model construction.

In validation Cohort 1 (Fig. 11B), there were significant variations in AUC between threshold_0.60 and threshold_0.95 (*p* = 0.012), as well as threshold_0.70 and threshold_0.95 (*p* = 0.027). These results indicate that threshold_0.95 outperformed threshold_0.60 and threshold_0.70 in this external validation cohort. There were no significant difference between threshold_0.60 and threshold_0.70, or threshold_0.80 and threshold_0.95, showing that performance was partially overlapping across model subsets. In validation cohort 2 (Fig. 11C), the performance disparities amongst the models became more significant. Significant differences were found between threshold_0.80 and threshold_0.60 (*p* = 0.018), as well as between threshold_0.95, threshold_0.60, and threshold_0.70 (*p* < 0.05). Notably, threshold_0.95 revealed sustained dominance across comparisons, demonstrating its strong discriminative performance in unseen data.

**Fig. 12:**
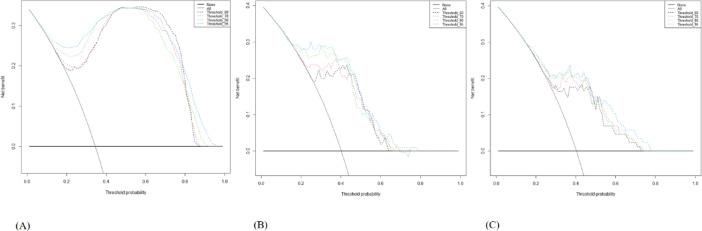
Decision curve analysis (DCA) of the predictive models. (**A**) Training cohort (**B**) Validation cohort 1 (**C**) Validation cohort 2. *Note: Threshold labels were expressed in percentage format (e.g.*,* “Threshold_95” corresponds to a threshold value of 0.95)*.

DCA was used to evaluate each model’s net clinical benefit across a range of threshold probabilities (Fig. 12, A–C). Two clinical choice extremes, treating all patients versus treating none, were used to compare the DCA curves. All models showed clinical utility across a wide range of threshold probabilities (about 10–90%) in the training cohort (Fig. 12A). Its potential for better clinical decision-making is demonstrated by threshold_0.95, which consistently displayed a somewhat higher net benefit, especially between threshold probabilities of 10% and 60%. There was a close alignment and relative similarity in the performance of threshold_0.60, threshold_0.70, and threshold_0.80.

In validation Cohort 1 (Fig. 12B), threshold_0.95 continued to provide the greatest net benefit across clinically meaningful thresholds. Threshold_0.60 and threshold_0.70 showed a reduced net benefit beyond the 50% threshold, indicating poorer precision in those areas. The decision curves for threshold_0.95 remained consistently above the “treat all” and “treat none” methods, demonstrating its practical utility in real-world implementation.

In validation Cohort 2 (Fig. 12C), threshold_0.95 and threshold_0.80 both had a substantial net benefit in this cohort, with threshold_0.95 slightly exceeding threshold_0.80 between 10% and 50%. Threshold_0.60 and threshold_0.70 demonstrated modest net benefit, especially at lower thresholds when early detection is crucial. These findings demonstrate threshold_0.95’s constant clinical relevance across diverse patient populations.

## Discussion

ALNM diagnosis in breast cancer patients is a critical healthcare challenge that necessitates precise and efficient approaches to provide timely identification and treatment. ALN dissection and SLN biopsy are currently the most commonly used procedures for ALN staging. However, both surgeries have varied levels of postoperative complications and morbidity. Recent research has focused on reducing the needless axillary procedures and preventing the overtreatment of breast cancer.

In our study, we successfully developed a US-based GCN model to assess ALNM preoperatively for breast cancer patients. The model performed satisfactorily in all the external validation cohorts, with AUCs of 0.88 (0.81–0.94) and 0.84 (0.74–0.92) and accuracies of 0.76 (95% CI: 0.68–0.84) and 0.74 (95% CI: 0.64–0.83) in validation cohorts 1 and 2, respectively. This is a promising strategy for predicting ALNM and avoiding unnecessary axillary therapy. Notably, the relational edge table contains several connections (edges) between the nodes. The existence of so many edges might introduce noise, redundancy, potentially irrelevant information, and increased complexity into the model^41^.

In this work, we considered different cosine similarity correlation threshold values for node connections to reduce the number of edges and improve the model’s robustness. Notably, increasing the threshold values reduces the number of edges while improving performance. For large threshold values, the nodes have a high correlation, which enhances accuracy.

To analyze and compare the predictive performance of our independently trained models, each generated using a different threshold (0.60, 0.70, 0.80, and 0.95), ANOVA was used. Significant differences in model performance were seen in both the validation cohort 1 (F(3, 333) = 24.28, *p* < 0.0001) and the external validation cohort 2 (F(3, 258) = 18.93, *p* < 0.0001). These data suggest that the models’ ability to predict ALNM changes with the threshold employed during model development.

In this study we demonstrated a high level of agreement between the four predictive models in both the training and validation cohorts. In the training cohort, model predictions were nearly perfectly correlated, with Pearson correlation coefficients more than 0.95 for all pairwise comparison. This strong alignment demonstrates the resilience of our modeling approach despite different threshold settings.

Validation cohort 1 retained similarly significant inter-model correlations, but slightly lower than the training cohort, indicating predicted variability when applied to new data. Notably, correlations between models with neighboring thresholds (e.g., threshold_60 vs. threshold_70) were very high (> 0.97), indicating robust predictive behavior despite small threshold changes.

In contrast, validation cohort 2 showed more variability, especially between models with the most divergent thresholds (threshold_60 vs. threshold_95, *r* = 0.87). This decreased correlation could imply increasing variability in the external dataset or the susceptibility of model outputs to threshold selection in various clinical scenarios. However, correlations between models with tighter threshold values remained in the upper 0.90s.

Overall, the findings reveal that, while predictive models are highly consistent across cohorts and threshold settings, slight divergence occurs with more extreme threshold differences and external validation.

The DeLong test findings (Fig. 11) reveal that, while all four models perform equally in the training cohort, substantial differences in AUC appear in the validation cohorts, with threshold_0.95 demonstrating higher generalizability. These findings indicate that performance criteria based exclusively on training data may obscure genuine differences in model robustness and discrimination.

To support these findings, the DCA (Fig. 12) demonstrates that threshold_0.95 consistently provides the largest net clinical benefit across all relevant threshold probabilities in all three cohorts. This strengthens the idea that threshold_0.95 not only performs well statistically, but also has superior practical utility when used in clinical decision-making.

Interestingly, threshold_0.80 has a significant decision support value in validation cohort 2, indicating that it may be an alternative in certain scenarios, but slightly less consistent than threshold_0.95.

These findings show that evaluating models based on both statistical significance (by DeLong tests) and clinical utility (using DCA) provides a more complete assessment of model suitability for deployment. Threshold_0.95 emerges as the most promising candidate for real-world integration, according to its excellent discrimination and constant high net benefit across datasets.

A deep learning radiomic nomogram was created by Zheng et al.^23^. with an AUC of 0.902 to predict ALNM in patients with early-stage breast cancer. Using clinical data and standard US, Wei et al.^42^ built a deep learning radiomic model. They reported an AUC of 0.819 for classifying low-load from heavy-load ALNM and 0.920 for classifying ALN-negative from ALN-positive cases. Using deep learning and combined radiomics models based on MRI and mammography, Guo et al.^43^ found that their best-performing model (a combined MLP) had an AUC of 0.846.

Chen et al.^44^ used DenseNet121 to extract deep learning features from DWI-ADC and DCE-MRI, which they then integrated with clinicopathological parameters to create a nomogram with AUCs of 0.80 (training) and 0.71 (testing) for ALNM prediction. Chihao et al.^45^ developed DAMF-former, a human-AI collaboration framework that uses dual-modal US elastography images and has an AUC of 0.883, greatly improving the diagnostic accuracy of junior radiologists. Wang et al.^46^ used ensemble learning to create a multiparametric MRI model that included T1WI, T2WI, and DWI features, resulting in an AUC of 0.996 for ALNM predictions. In contrast, our US-based GCN model, created utilizing a smaller, independent dataset, achieved a high AUC of 0.88 for ALNM prediction. Unlike earlier studies that used large-scale datasets, high-resolution multimodal imaging (e.g., MRI, mammography), or complex fusion and ensemble procedures, our model relies just on traditional US-derived deep learning features and a graph-based learning framework. Despite its simplicity, the model outperformed other multimodal or deep fusion approaches. This demonstrates our method’s practicality, computational efficiency, and real-world deployment potential, especially in resource-constrained clinical settings where traditional US is the only option.

The US-based GCN model was validated via two external validation cohorts. Furthermore, to the best of our knowledge, this is the first study to create a GCN model based on US deep learning features to predict ALN status in patients with breast cancer, and the results are encouraging.

Although the study’s GCN-based strategy produced promising results, there are a few issues that can be addressed in future investigations. The model’s robustness could be improved with a larger dataset. Patients with multifocal breast lesions and bilateral disease were excluded because it was difficult to determine which lesion would lead to ALN metastases and should be input into the model. As a result, the current model can predict ALNM only for patients with a single type of breast cancer. Additional research is needed to develop another model to predict ALNM status for patients with multifocal breast lesions and bilateral disease.

In summary, we established a US-based GCN model to evaluate ALN status preoperatively in patients with unifocal breast cancer. To the best of our knowledge, this is the first study to build a GCN model based on US deep learning features to predict ALN status in patients with breast cancer. The US-based GCN model is noninvasive and feasible for predicting ALNM in breast cancer patients.

## Data Availability

The data that support the findings of this study are available from the corresponding author upon reasonable request. The code for the study is available at Enock-Agyekum/GCN-Based-Axillary-Lymph-Node-Metastasis-Prediction-in-Breast-Cancer-Patients.
